# Accuracy of the surgical execution of virtually planned deep circumflex iliac artery flaps and their appropriateness for masticatory rehabilitation

**DOI:** 10.1186/s13005-024-00444-y

**Published:** 2024-08-13

**Authors:** Florian Peters, Stefan Raith, Anna Bock, Kristian Kniha, Mark Ooms, Stephan Christian Möhlhenrich, Frank Hölzle, Ali Modabber

**Affiliations:** 1 Department of Oral, Maxillofacial and Facial Plastic Surgery, University Hospital RWTH Aachen, Pauwelsstr. 30, 52074 Aachen, Germany; 2 Department of Orthodontics, University Witten/Herdecke, Private Universität Witten/Herdecke GmbH, Alfred-Herrhausen-Straße 45, 58448 Witten, Germany

**Keywords:** Deep circumflex iliac artery flap, DCIA, Virtual surgery planning, Computer-aided design, Free flaps

## Abstract

**Background:**

Tumorous diseases of the jaw demand effective treatments, often involving continuity resection of the jaw. Reconstruction via microvascular bone flaps, like deep circumflex iliac artery flaps (DCIA), is standard. Computer aided planning (CAD) enhances accuracy in reconstruction using patient-specific CT images to create three-dimensional (3D) models. Data on the accuracy of CAD-planned DCIA flaps is scarce. Moreover, the data on accuracy should be combined with data on the exact positioning of the implants for well-fitting dental prosthetics. This study focuses on CAD-planned DCIA flaps accuracy and proper positioning for prosthetic rehabilitation.

**Methods:**

Patients post-mandible resection with CAD-planned DCIA flap reconstruction were evaluated. Postoperative radiograph-derived 3D models were aligned with 3D models from the CAD plans for osteotomy position, angle, and flap volume comparison. To evaluate the DCIA flap’s suitability for prosthetic dental rehabilitation, a plane was created in the support zone and crestal in the middle of the DCIA flap. The lower jaw was rotated to close the mouth and the distance between the two planes was measured.

**Results:**

20 patients (12 males, 8 females) were included. Mean defect size was 73.28 ± 4.87 mm; 11 L defects, 9 LC defects. Planned vs. actual DCIA transplant volume difference was 3.814 ± 3.856 cm³ (*p* = 0.2223). The deviation from the planned angle was significantly larger at the dorsal osteotomy than at the ventral (*p* = 0.035). Linear differences between the planned DCIA transplant and the actual DCIA transplant were 1.294 ± 1.197 mm for the ventral osteotomy and 2.680 ± 3.449 mm for the dorsal (*p* = 0.1078). The difference between the dental axis and the middle of the DCIA transplant ranged from 0.2 mm to 14.8 mm. The mean lateral difference was 2.695 ± 3.667 mm in the region of the first premolar.

**Conclusion:**

The CAD-planned DCIA flap is a solution for reconstructing the mandible. CAD planning results in an accurate reconstruction enabling dental implant placement and dental prosthetics.

## Background

Oral squamous cell carcinoma and other tumours of the jaw have a rising incidence. In some cases, continuity resection is the only way to cure the patient. After this procedure, patients have a limited quality of life. Without reconstruction of the defect, the patients suffer from severe impairments that cause significant restrictions [1, 2]. The reconstruction of the mandible with a microvascular bone flap is the actual gold standard in the literature [3]. Deep circumflex iliac artery (DCIA) flaps and free fibula flaps are commonly used for bony reconstruction of the jaw. Nowadays, computer aided planning (CAD) is widely used for reconstructing the mandible. For this purpose, computed tomography (CT) images are used and then converted into three-dimensional (3D) models. The bony flap is fitted into the defect and cutting guides are designed. The cutting guides are used to transfer the planning into the operation theatre. This CAD planning should lead to more accuracy in the reconstruction, satisfactory aesthetic outcomes and reduction in the rate of complications [4–7]. Additionally, the dental prosthetic rehabilitation can be considered during the transplant’s CAD planning. By aligning the position of the bone flap to the planned dental prosthetic rehabilitation, restauration can be achieved and quality of life improved.

The actual achievable accuracy of using CAD reconstruction planning has been evaluated in numerous studies on the free fibula flap [8–13]. However, data on the accuracy achievable by CAD-planned DCIA flaps is scarce. Moreover, data on accuracy should be combined with data on the exact positioning of the implants to ensure well-fitting dental prosthetics.

Therefore, the aim of this study was to evaluate the achievable accuracy of CAD-planned DCIA flaps. Furthermore, the flap’s positioning and suitability for subsequent prosthetic rehabilitation using dental implants should be assessed to investigate whether CAD planning of the DCIA flap facilitates dental rehabilitation.

## Methods

After institutional approval (EK294-20), all the patients who received a reconstruction of the mandible with a CAD-planned DCIA flap from 2012 to 2021 were reviewed. Inclusion criteria was a continuity resection of the mandible. The reconstruction with a DCIA flap was planned computer aided using ProPlan CMF Version 1.1 to Version 3.01 (Materialise, Leuven, Belgium). After performing the DCIA flap, a 3D radiograph of the patient had to be available and of acceptable quality.

### Collection of data

After finding a patient eligible for this study the age, sex, the diagnoses responsible for the mandible resection and the donor site of the DCIA flap were collected.

The postoperative radiographs of these patients were downloaded as Digital Imaging and Communications in Medicine (DICOM) files from the local Picture Archiving and Communication System (PACS). The 3D models of the preoperative planning were exported from the planning software ProPlan CMF 3.01.

### Analysis of planning

The postoperative 3D radiograph was imported into ProPlan CMF 3.01 and the mandible, the maxilla as well as the DCIA transplant were segmented. The generated 3D models and the preoperative models were imported to Geomagic Control 2014 (3D Systems Corporation, Rock Hill, SC, USA). The postoperative models of the DCIA transplant were aligned with the model of the preoperative planned DCIA transplant via the Best Fit algorithm of Geomagic Control.

**Fig. 1:**
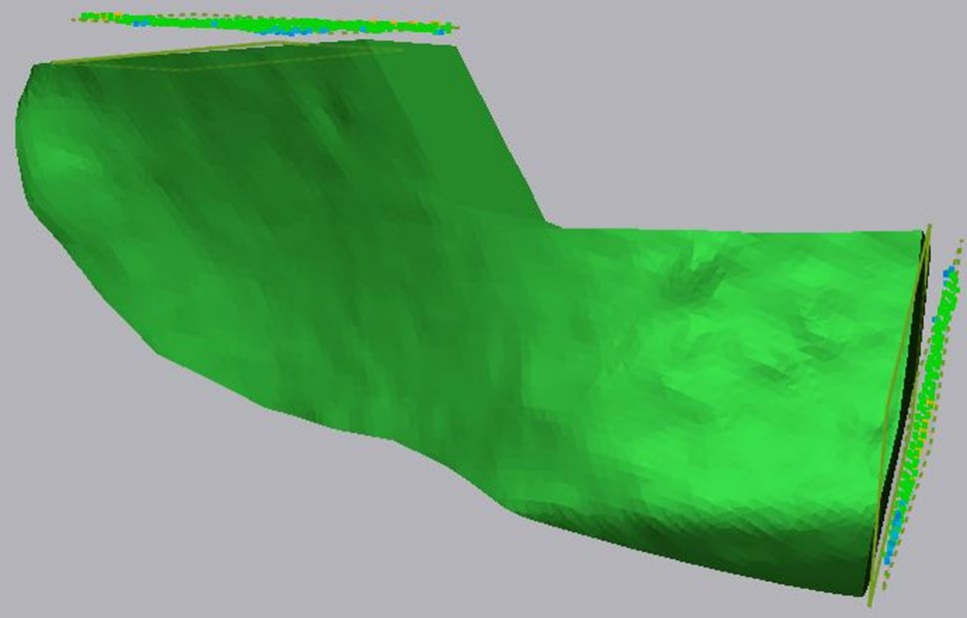
Three-dimensional model of a DCIA flap with equalisation planes created at the osteotomies ventral and dorsal. The equalisation planes were averaged through all points of the dorsal or ventral surfaces of the DCIA flap. The postoperative DCIA flap is invisible, but its equalisation planes are shown with the dashed outline and the coloured points in them

**Fig. 2:**
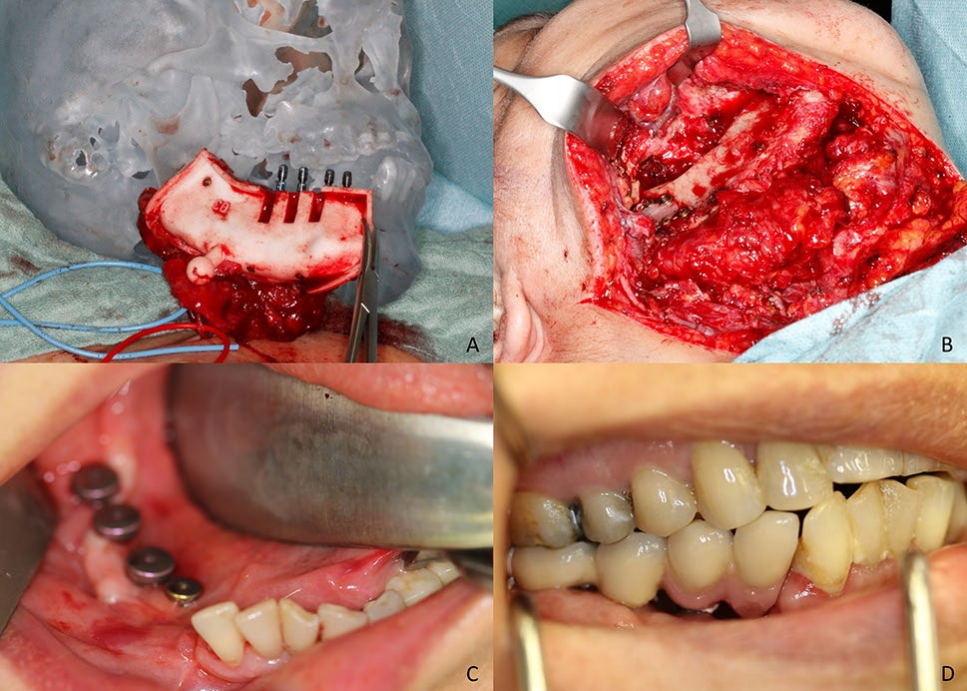
**A** Raised DCIA flap with cutting guide attached to it. In this case the dental implants have been inserted while the flap was still attached to the vessels of the pelvis. **B** DCIA flap anastomosed to the cervical vessels and mounted to the lower jaw with osteosynthesis plates. **C** Dental implants after uncovering intraoral. **D** Final dental prosthesis attached to the dental implants

After the aligning, equalisation planes were drawn at the ventral and dorsal osteotomies of the DCIA flap model from the preoperative planning and the follow-up radiograph (Fig. 1). Between the equalisation planes at each osteotomy of the model, the difference in distance and in the angle of the osteotomies were determined. Furthermore, the volume of the planned flap and the postoperative DCIA flap model were compared (Fig. 2).

**Fig. 3:**
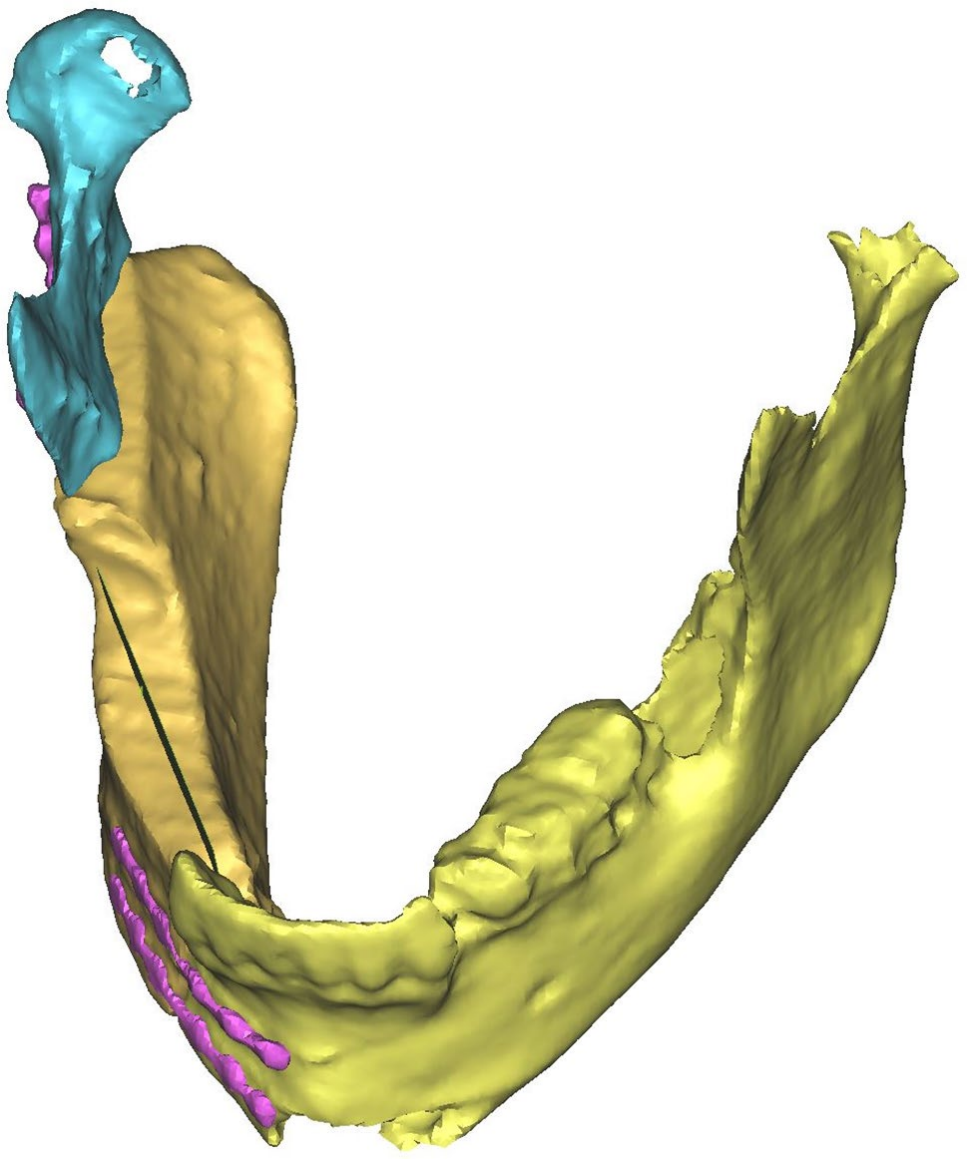
Three-dimensional model of a reconstructed mandible with aplane placed exactly in the middle of the deep circumflex iliac crest transplant used as reference to the position of the upper teeth

**Fig. 4:**
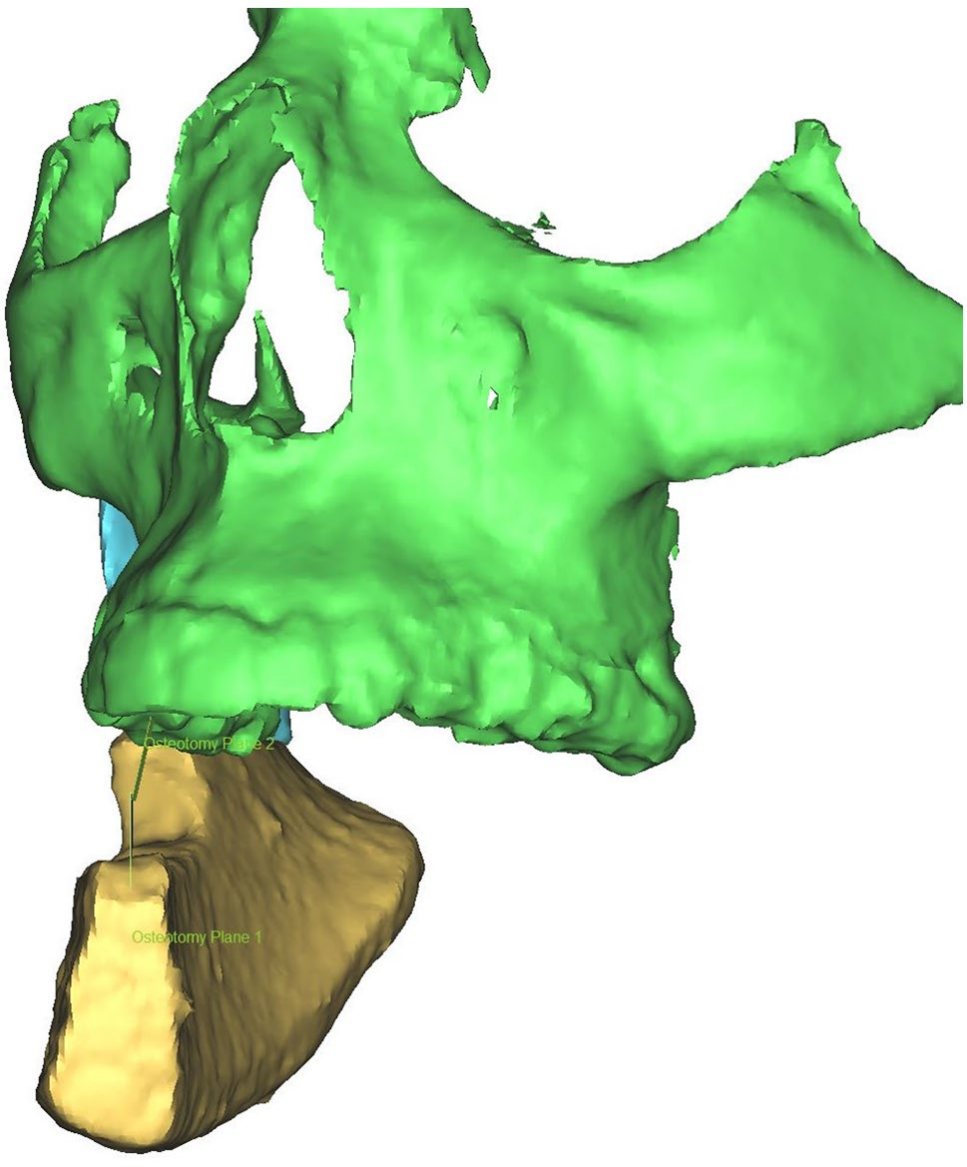
Position of the osteotomy plane from the upper jaw and from the lower jaw as they relate to each other in occlusion. The linear distance between them was measured. The upper jaw is hidden for clarity reasons

To evaluate the patient’s suitability for prosthetic dental rehabilitation, an osteotomy plane was created in ProPlan 3.01. The extend was 1 × 5 × 0.1 mm. This plane was placed in the fissure of the two premolars on the side where transplant was placed. The axis of the plane was aligned to the axis of the two premolars in the upper jaw to represent the location of a support zone defined by Eichner [14]. On the top of the DCIA transplant, the middle was measured at two different points. An osteotomy plane was placed in the middle of the top’s surface and aligned to the axis of the DCIA transplant (Fig. 3). Then the DCIA transplant was virtually cut at this osteotomy plane. For testing the position of the DCIA transplant in relation to the upper jaw, the DCIA transplant and the rest of the jaw were rotated along the rotation centre of the temporomandibular joint. After hiding the maxilla, the lateral distance between the dental axis of the upper jaw and the DCIA transplant could be measured (Fig. 4).

### Surgical procedure

A consultant in the department found that every patient was eligible for mandible reconstruction using a DCIA flap. A CT scan was performed on every patient of the head and neck areas as well as the pelvis. Using ProPlan CMF, a 3D model of the maxilla, mandible and the pelvis was segmented. Then, the later position of the osteotomies was chosen. A piece of iliac crest was chosen and fitted inside the defect. Cutting guides for the mandible and the iliac crest were manufactured from Polyamide using a selective laser sintering 3D printer at Materialise (Materialise, Leuven, Belgium).

In the operation theatre the mandible and the iliac crest were exposed. The cutting guides were mounted to the bone and fixed by 2.0 osteosynthesis screws. The bone was cut using an oscillating saw. After sawing the DCIA flap, the miniplates were pre-bent using a model of the mandible and were fixed to the DCIA flap using 2.0 osteosynthesis screws. Two miniplates were used at the ventral end and two were used at the dorsal end. Then, the DCIA flap was transferred to the mandible and fixed there using 2.0 osteosynthesis screws. After fixation the microvascular anastomoses were performed with a surgical microscope to the A. thyroidea superior or the A. facialis at the throat. The venous anastomosis was performed end to side at the V. jugularis interna.

### Statistical evaluation

All statistical tests were performed using the Prism GraphPad Version 9 (GraphPad Software Inc., San Diego, CA, USA). The data were checked for normal distribution with the D’Agostino-Pearson normality test in the K2 variant. Further analyses were performed using a t-test with Welch’s correction. The level of significance was *p* ≤ 0.05. All data were expressed as mean values ± standard deviation.

## Results

The inclusion criteria were met by 20 patients who received a computer-planned DCIA flap after continuity resection of the mandible. Of these, 12 patients were males and eight females. The mean age was 75.64 ± 31.06 years. From the whole collective, three patients received adjuvant radiotherapy. The mean defect size was 73.28 ± 4.87 mm and 11 were classified as an L defect. The other nine patients had an LC defect as defined by Jewer, Boyd [15]. Twelve of the mandibular defects were on the left side. The diagnoses that made the resection necessary are displayed in Table 1. The DCIA flap was taken from the right or the left iliac crest in 10 cases each. Out of 20 patients, 10 had all their teeth left besides the area of the reconstructed defect, four patients lost one or more molars on both sides and six patients were completely edentulous. A primary reconstruction had been performed in 12 cases and eight had secondary reconstructions. Twelve patients (60%) were rehabilitated with dental implants. All implants placed could be used for dental prosthetics. The mean duration between the operation and the postoperative scan of the patient was 125.1 ± 138.3 days.

**Table 1:** This table shows the epidemiological data of the patient collective

Sex	
female	8
male	12
**Affected side**	
right	8
left	12
**Adjuvant radiotherapy**	
yes	3
no	17
**Kind of reconstruction**	
primary	12
secondary	8
**Diagnosis**	
Ameloblastoma	4
Squamous cell carcinoma	5
Osteomyelitis	7
Sarcoma	1
Myxoma	1
Keratocyst	1
Haemangioma	1
n	20

**Fig. 5:**
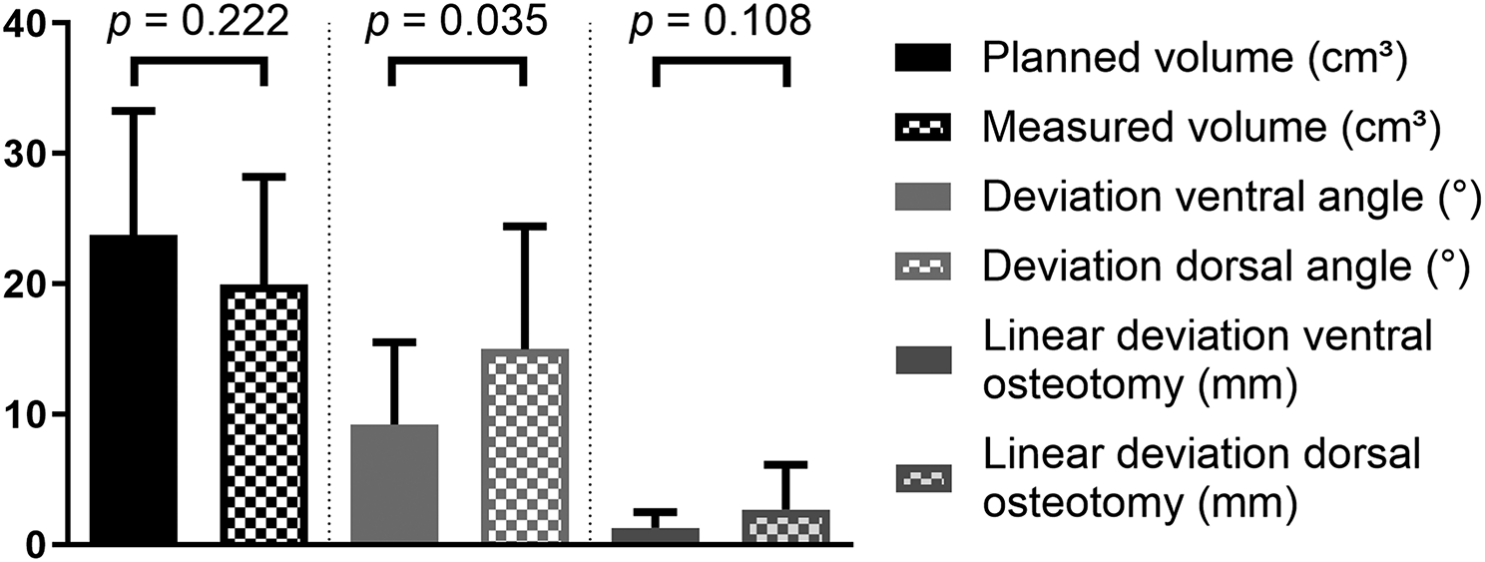
Boxplot showing the planned and measured volume of the DCIA flap, deviation between the planned and actual angle of the osteotomies as well as the deviation between the planned and actual linear position of the osteotomies of the DCIA transplant

The difference in volume between the planned DCIA transplant and the actual DCIA transplant was 3.814 ± 3.856 cm³ in mean. The mean volume for the planned DCIA transplant was 23.754 ± 9.530 cm³. The mean volume of the actual DCIA transplant was 19.941 ± 8.292 cm³. The difference in volume between the planned and the actual DCIA transplants was not statistically significant (Fig. 5).

The difference of the dorsal osteotomy angle between the planned and actual DCIA flap was 14.97 ± 9.433°. The difference of the ventral osteotomy angle was 9.216 ± 6.303°. The deviation from the planned angle was significantly larger at the dorsal osteotomy than at the ventral osteotomy (Fig. 5). There was no significant difference in the deviation of the dorsal osteotomy (*p* = 0.9314) or the ventral osteotomy (*p* = 0.6038) when the DCIA was placed on either the left or right side of the mandible. Additionally, no significant differences in the deviation of the dorsal (*p* = 0.849) and ventral (*p* = 0.6868) osteotomy could be measured when comparing patients with benign lesions (primary reconstruction) to those with malignant lesions (secondary reconstruction).

The measured linear difference between the planned DCIA transplant and the actual DCIA transplant from the follow-up radiograph were 1.294 ± 1.197 mm for the ventral osteotomy and 2.680 ± 3.449 mm for the dorsal osteotomy. The difference was statistically not significant (Fig. 5).

The difference between the dental axis and the middle of the DCIA transplant ranged from 0.2 mm to 14.8 mm. The mean lateral difference was 2.695 ± 3.667 mm in the region of the first premolar.

## Discussion

At present, resection of the mandible due to conditions such as oral squamous cell carcinoma, osteomyelitis, or ameloblastoma remains state-of-the-art [16–18]. In severe cases, a continuity resection of the mandible is inevitable, leading to a reduced quality of life for these patients [19]. Bony reconstruction of the mandible not only improves their quality of life but also represents the current gold standard of treatment [1, 20]. Complete dental rehabilitation, achievable after a microvascular bone graft, further enhances their quality of life [21].

The free fibula flap is commonly used for microvascular bony reconstruction of the mandible [22], offering advantages such as significant bone length and low donor site morbidity. However, its bone height is limited, making the insertion of dental implants and masticatory rehabilitation challenging [23]. When the defect involves the angle of the jaw, the DCIA flap presents an optimal contour for reconstruction of this part of the mandible. It provides sufficient bone to reconstruct half of the mandible, with ideal vertical bone height for dental implant placement [24].

In contemporary microvascular reconstruction of the mandible, CAD-planning is used in almost all cases. Previous research indicates that CAD-planning leads to advantages in accuracy, shorter operative and ischemic times for the transplant compared to conventional planning [8, 10, 12]. The majority of these comparisons have been conducted on CAD-planned free fibula flaps. However, the evaluation of the achievable accuracy of the CAD planning for the DCIA flap is scarce in the literature [25]. Existing studies on this topic often involve very few cases [5, 26] with them mostly examine parameters that do not reflect the accuracy of the planning [27, 28].

This study’s focus was to evaluate the accuracy achieved by CAD planning of DCIA flaps and their positioning for subsequent dental rehabilitation of the patients. Therefore, preoperative planning models were compared to the postoperative models obtained from the patient’s postoperative CT scans. The volume of the models, as well as the position and angulation of the osteotomies on the DCIA transplant, were compared. For these measurements, the surface of the osteotomised side of the DCIA flap was marked. An equalisation plane was created using Geomagic Control. These equalisation planes enabled the measurement of the position and angle of the osteotomy, averaged over the entire surface of the osteotomy. Measuring only the linear deviation between the planned and actual osteotomies at the buccal side of the mandible would not detect any deviations on the lingual side or in the cutting angle.

Some of the patients were not in occlusion during the postoperative X-ray. Therefore, occlusion was later achieved by virtually rotating the mandible around the rotation centre of the temporomandibular joints. This method was applied for both edentulous and dentate patients as an uniform approach. Adjusting the occlusion through the teeth in the postoperative X-rays was not possible due to frequent artefacts caused by dental prostheses. The fit of the planning for the subsequent dental rehabilitation was assessed. For this purpose, the tooth axis of the maxillary teeth was marked in the middle of the posterior tooth row at the level of the first premolar. The centre of the DCIA flap needed to align precisely with this spot to simplify dental rehabilitation using dental implants. This area was chosen because it is the anterior support zone defined by Eichner [14]. Rehabilitation of this support zone is important for dental rehabilitation. The anterior support zone was selected because, after microvascular reconstruction, implants are often placed more anteriorly as handling and cleaning them becomes more challenging the further dorsally they are placed. With a mean lateral shift of 2.695 ± 3.667 mm, dental rehabilitation should easily be feasible. This should be comparable to the advantages for dental rehabilitation after using free fibula flaps [29]. In the studies performed by Shu and Liu [26], Zhang and Yu [28], the positioning of the DCIA flap with regard to the later insertion of dental implants was not addressed. This, as the patient’s most important parameter, should be considered in every CAD planning of dental rehabilitation. Only adequate dental rehabilitation significantly increases the patient’s quality of life in microvascular osseous reconstruction. From this study’s cohort, 60% (*n* = 12) of the patients received dental implants supporting dental prosthetics. The positioning of the dental implants and the DCIA flap was sufficient for the dental rehabilitation of these patients. The patients who did not receive dental implants either chose not to have them (*n* = 6; 30%) or suffered a recurrence of their underlying disease (*n* = 2; 10%).

The condyle’s position has been evaluated in several other studies as an indicator of the reconstruction’s accuracy [27, 28]. Even though the condyle is the last part of the mandible and could indicate inaccuracies due to positional shifts, the measurement method is not sufficient. If only lateral displacement is measured [27], then caudal and cranial displacements are ignored. Other measurement methods that attempt to measure displacement in all directions show a heatmap of the displacement, but do not provide actual values [28].

In this study, the osteotomies had a lateral displacement of 1.294 ± 1.197 mm for the ventral osteotomy and 2.680 ± 3.449 mm for the dorsal osteotomy. Compared to a lateral displacement of the osteotomy of 0.70 ± 0.16 mm for the anterior and 1.47 ± 0.37 mm for the dorsal osteotomy, as measured by Zhang and Yu [28]. Shu and Liu [26] measured the displacement of the osteotomy line on the mandible in general and revealed a displacement of 2.06 ± 0.86 mm, a value within the same range as this study. In contrast to this study, those conducted by Zhang, Yu [28] and Shu, Liu [26] included only patients with benign lesions. For malignant diseases, a larger resection is necessary, which can lead to a greater deviation of the osteotomies. However, the results of this study showed no significant difference in the deviation between the planned and actual osteotomy (dorsal osteotomy *p* = 0.849; ventral osteotomy *p* = 0.6868) comparing malignant and benign cases. This lack of significance might be due to the small sample size of this study or it could indicate that the type of disease has no significant impact on the accuracy of the planned DCIA flap implementation.

The mean defect size in the present study was 73.28 ± 4.87 mm compared to 62.1 ± 9.6 mm in the study by Zhang and Yu [28]. Shu and Liu [26] did not report any defect size in their study. Furthermore, all patients treated in the studie by Shu and Liu [26] (*n* = 8) and Zhang and Yu [28] (*n* = 45) received a primary reconstruction. In the present study, 40% (*n* = 8) of the patients received a secondary reconstruction, which can lead to a greater deviation of the osteotomies. A secondary reconstruction is technically more challenging due to the existing scarring that must be loosened. The scarring can cause a deviation of the jaw that needs to be corrected during the operation. Furthermore, artefacts in the CT scan used for computerized planning artefacts can be caused by the osteosynthesis plates, making the placement of the cutting guide less accurate.

Additionally, Shu and Liu [26] did not provide any information on the osteosynthesis. Another reason for the slightly greater displacement in the present study could be the use of intraoperative navigation by Zhang and Yu [28]. While the cutting guides used in the present study produce a small error, intraoperative navigation could reduce the error and the lateral displacement. The deviation of the osteotomy angle was significantly higher (*p* = 0.035) for the dorsal osteotomy than for the ventral one. The greater inaccuracies at the dorsal osteotomy line could be explained by the fact that the dorsal osteotomy is more difficult in terms of surgical skill, as the space conditions there are significantly more limited. The planned and the actually measured volumes of the DCIA transplant differed by 3.814 ± 3.856 cm³. This volume difference results from inaccuracies, shaping, and resorption. A volume difference of 2094.35 ± 929.12 mm³ was measured between the planned and the actual shaped DCIA transplant by Shu and Liu [26]. The volume difference in this study is higher than what Shu and Liu [26] measured. The reason could be the period of time between the operation and the postoperative radiograph. With 14 days the period between the operation and the postoperative radiograph was short in the study of Shu and Liu [26]. The resorption rates for microvascular DCIA flaps are 3% over two years [30]. When dental implants are inserted, the resorption in the area of the implants is higher than in the rest of the bone [31]. However, even a mean time of 125 days to the postoperative scan cannot fully explain the volume loss measured in this study by resorption only. The shaping of sharp edges during the reconstruction with the DCIA flap is also responsible for the excess volume loss.

## Conclusion

The CAD-planned DCIA flap is an effective solution for reconstructing the mandible, providing a high amount of bone that simplifies dental rehabilitation. CAD planning results in accurate reconstruction facilitating the placement of dental implants and subsequent dental prosthetics. However, comparing the accuracy achieved to the literature is challenging due to the variety of parameters evaluated in different studies.

## Data Availability

The data is available from the corresponding author on reasonable request.

## Abbreviations

CAD: Computer aided design
3D: Three-dimensional
CT: Computerized tomography
DCIA: Deep circumflex iliac artery
